# S100A4–TLR4–TGF-β axis as a therapeutic target for Dupuytren’s contracture in diabetic patients

**DOI:** 10.1038/s41420-026-03167-y

**Published:** 2026-05-23

**Authors:** Koki Kato, Shingo Komura, Yuta Yanagihara, Noritaka Saeki, Atsushi Goto, Rie Maki, Hitoshi Hirose, Akihiro Hirakawa, Yuuki Imai, Haruhiko Akiyama

**Affiliations:** 1 Department of Orthopaedic Surgery, Gifu University Graduate School of Medicine, Gifu, Japan; 2 Division of Integrative Pathophysiology, Proteo-Science Center, PIAS, Ehime University, Ehime, Japan; 3 Research Coordination and Technical Development Office, PIAS, Ehime University, Ehime, Japan; 4 Center for One Medicine Innovative Translational Research (COMIT), Gifu University, Gifu, Japan

**Keywords:** Mechanisms of disease, Skin diseases

## Abstract

Dupuytren’s contracture is a superficial fibrotic disease of the hands that causes flexion contractures of the affected fingers. Diabetes mellitus (DM) is a risk factor for Dupuytren’s contracture. However, the exact underlying mechanisms by which DM is involved in its development and progression remain unknown. This study investigated the involvement of glycometabolic disorders in the pathogenesis of Dupuytren’s contracture. RNA sequencing revealed that S100A4 expression was significantly increased in Dupuytren’s contracture-derived fibroblasts under high-glucose conditions compared with low-glucose conditions, and this finding was confirmed by immunoblotting and enzyme-linked immunosorbent assay. *S100A4* expression in Dupuytren’s contracture tissues was significantly higher in patients with diabetes than in those without. S100A4 was expressed in several cell types, including fibroblasts, myofibroblasts, and macrophages. However, the expression of its receptor, Toll-like receptor 4 (TLR4), was predominantly detected in CD68-expressing macrophages. Furthermore, recombinant S100A4 treatment significantly increased transforming growth factor-beta 1 (TGF-β1) expression, which is a central mediator of fibrosis, in macrophages. Pharmacological inhibition of TLR4 suppresses TGF-β1 upregulation via S100A4. Thus, the S100A4–TLR4–TGF-β axis could be a potential therapeutic target for Dupuytren’s contracture in diabetic patients.

## Introduction

Dupuytren’s contracture, which is caused by genetic, epigenetic, and environmental factors, is a superficial fibrotic disease that develops in the hands. This disease causes flexion contractures of the affected fingers and impacts on hand function, quality of life, and health burden [1]. Although a collagenase Clostridium histolyticum injection has been developed as a minimally invasive therapy, surgical excision is still a standard treatment for this disease [2]. However, surgical complications, including nerve injuries and delayed wound healing, have been reported, with incidences of 23% [3]. Moreover, these current treatments have high recurrence rates, highlighting the need for mechanistic insights.

Diabetes mellitus (DM) is a risk factor for Dupuytren’s contracture [4, 5]. A prospective observational study revealed that the prevalence and cumulative incidence of Dupuytren’s contracture are higher in patients with diabetes than in those without (prevalence: 15.5% vs. 5.6%; 4-year cumulative incidence: 13.4% vs. 9.1%) [6]. A possible mechanism between DM and Dupuytren’s contracture has been proposed, which includes increased levels of advanced glycation end products (AGEs) and collagen glycation [7, 8]. However, the exact underlying mechanisms by which DM is involved in the development and progression of Dupuytren’s contracture remain unclear.

*S100A4*, also called fibroblast-specific protein 1 (*FSP1*), is a member of the S100 family of proteins with calcium-binding domains [9, 10]. S100A4 functions intracellularly and extracellularly and is expressed in various cell types, including fibroblasts, myofibroblasts, macrophages, and vascular cells [9, 10]. It is known as a damage-associated molecular pattern (DAMP) that is released upon cell damage, death, or stress. Extracellular S100A4 has been implicated in fibrosis, including kidney, liver, pulmonary, and systemic sclerosis in mice and humans [9, 11, 12]. S100A4 binds to its receptors, including receptor for advanced glycation end product (RAGE) and Toll-like receptor 4 (TLR4) [11, 13, 14] and regulates cell motility, invasion, and the production of proinflammatory cytokines and chemokines through RAGE and TLR4 signalling [11]. Recently, stromal cell–immune cell interactions have been identified as a potential mechanism for Dupuytren’s contracture development and progression [15–20]. However, the role of S100A4 in Dupuytren’s contracture has not been investigated, and its function in stromal cell–immune cell interactions in Dupuytren’s contracture remains unknown.

This study aimed to investigate the involvement of glycometabolic disorders in the pathogenesis of Dupuytren’s contracture. Dupuytren’s contracture-derived fibroblasts treated with high-glucose media showed upregulated *S100A4* expression compared to those treated with low-glucose media. *S100A4* mRNA expression levels in diseased tissues positively correlated with the HbA1c levels of the patients. The S100A4-positive area was larger in patients with DM than in those without. Furthermore, CD68-expressing (CD68^+^) macrophages in Dupuytren’s contracture tissues expressed TLR4. Recombinant S100A4 administration did not alter the cell migration and chemotaxis of human macrophages, whereas it significantly increased the expression of transforming growth factor-beta 1 (TGF-β1), a fibrotic mediator, in these cells. Finally, pharmacological TLR4 inhibition suppressed TGF-β1 upregulation via S100A4 in human macrophages.

## Results

### High-glucose treatment upregulates *S100A4* expression in Dupuytren’s contracture-derived fibroblasts

**Fig. 1:**
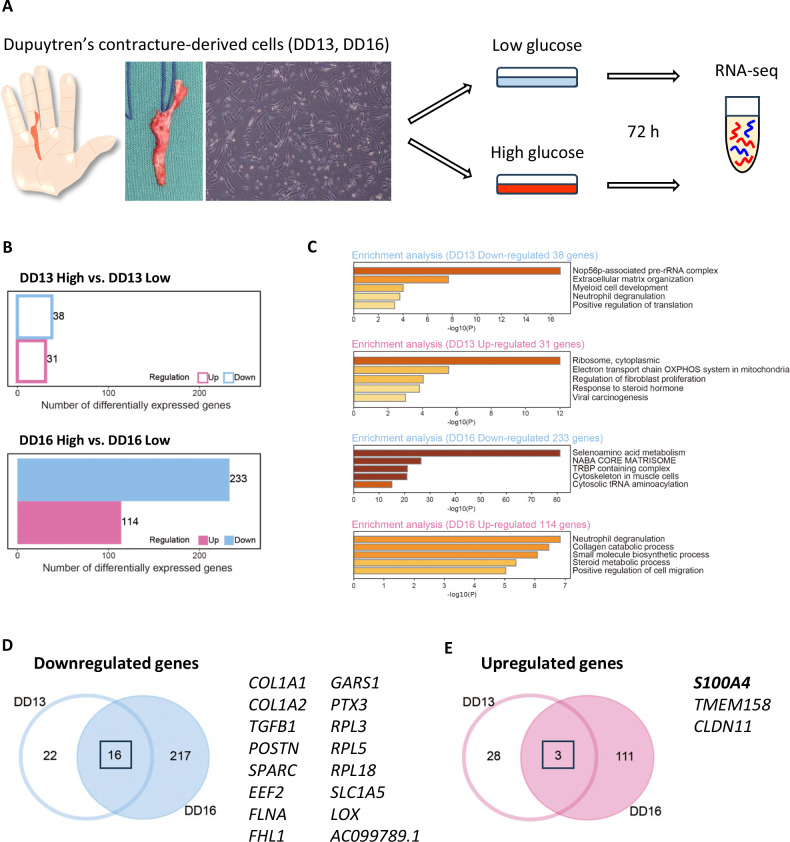
RNA sequencing analyses identified *S100A4*, which is upregulated by high-glucose treatment in Dupuytren’s contracture-derived fibroblasts. **A** Schematic representation of the RNA sequencing protocol. Dupuytren’s contracture-derived cells were harvested from two independent patients (DD13 and DD16). Cells were cultured in either low- or high-glucose medium for 72 h. RNA was extracted and analysed using RNA sequencing. **B** Number of differentially expressed genes (DEGs) in the DD13 and DD16 groups. Compared with low-glucose conditions, 31 and 38 genes were upregulated and downregulated, respectively, in DD13. In addition, 114 and 233 genes were upregulated and downregulated, respectively, in DD16. **C** Kyoto Encyclopaedia of Genes and Genomes (KEGG) pathway analyses for DD13 and DD16. **D** Venn diagram comparing the downregulated genes between DD13 and DD16. These analyses identified 16 commonly downregulated genes. **E** Venn diagram comparing the upregulated genes between DD13 and DD16. These analyses identified three commonly upregulated genes.

Dupuytren’s contracture-derived fibroblasts from two independent patients (DD13 and DD16) were cultured under high- and low-glucose conditions, and RNA sequencing analyses were performed to investigate whether glucose concentration affects Dupuytren’s contracture (Fig. 1A, Supplementary Fig. 1A, B). In DD13, 69 differentially expressed genes (DEGs) were identified, of which 38 and 31 genes were significantly downregulated and upregulated, respectively, under high-glucose conditions (Fig. 1B). In DD16, 347 DEGs were identified, and 233 and 114 genes were significantly downregulated and upregulated, respectively, under high-glucose conditions (Fig. 1B). Gene Ontology (GO) and Kyoto Encyclopaedia of Genes and Genomes (KEGG) pathway analyses revealed no shared terms between DD13 and DD16 (Fig. 1C). Moreover, principal component analysis revealed that the overall gene expression patterns varied more between patients than the differences observed between high- and low-glucose conditions (Supplementary Fig. 1C). DEGs shared between DD13 and DD16 were analysed. Notably, among the 16 shared genes downregulated under high-glucose conditions, fibrosis-associated genes, including *COL1A1*, *COL1A2*, *POSTN*, and *TGFB1*, were identified (Fig. 1D). These results indicate that high-glucose conditions may not directly induce fibrosis in diseased fibroblasts. In contrast, only three shared upregulated genes, including *S100A4*, *TMEM158*, and *CLDN11*, were identified in the high-glucose condition; *S100A4* was focused upon (Fig. 1E).

### DM is associated with increased *S100A4* expression in Dupuytren’s contracture tissues

**Fig. 2:**
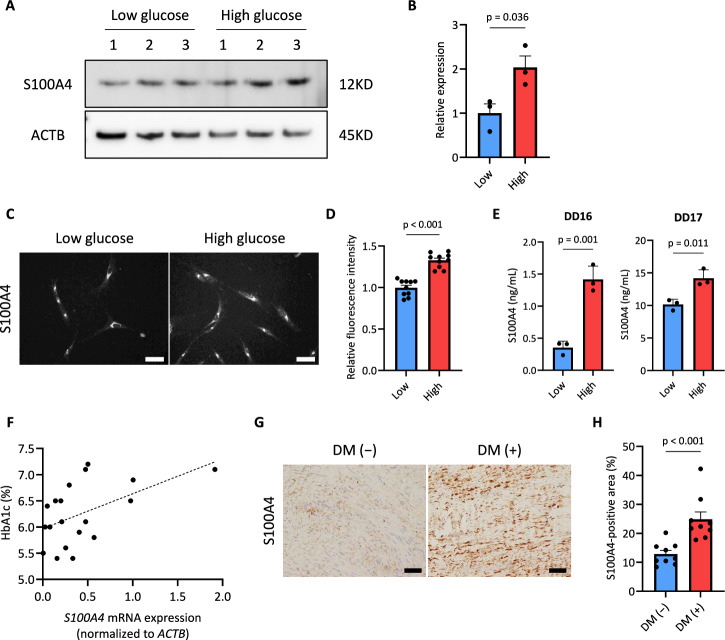
Diabetes mellitus is associated with increased S100A4 expression in Dupuytren’s contracture-derived fibroblasts and tissues. **A** Western blot analysis of Dupuytren’s contracture-derived fibroblasts cultured in either low- or high-glucose medium for 72 h. **B** Quantification of *S100A4* expression shown in Fig. 2A. S100A4 expression was higher under high-glucose conditions than under low-glucose conditions. *S100A4* expression was normalised to that of *ACTB*, and the mean value of the low-glucose condition was set to 1. Data are shown as the mean ± standard error of the mean (SEM) of three independent samples in each group (two-tailed Student’s t-test; *p* = 0.036). **C** Immunocytochemical staining of Dupuytren’s contracture-derived fibroblasts using an anti-S100A4 antibody was performed in either low- or high-glucose media for 72 h. **D** Quantification of the fluorescence intensity shown in Fig. 2C. The mean intensity in the low-glucose condition was set to 1. Data are shown as the mean ± SEM of 10 fields (two independent experiments) in each group (two-tailed Student’s t-test; *p* < 0.001). **E** Quantification of extracellular S100A4 levels (ng/mL). Culture media derived from Dupuytren’s contracture-derived fibroblasts (DD16 and DD17) treated with low or high glucose were analysed by enzyme-linked immunosorbent assay (biological triplicates for each condition). High-glucose treatment significantly increased extracellular S100A4 secretion (two-tailed Student’s t-test; DD16, *p* = 0.001; DD17, *p* = 0.011). **F** Correlation between *S100A4* mRNA expression and preoperative HbA1c in Dupuytren’s contracture tissues (DD1–DD19). *S100A4* expression was normalised to that of *ACTB*. A moderate positive correlation was observed (Pearson’s *r* = 0.52, *p* = 0.022). **G** Representative S100A4 immunohistochemical staining of Dupuytren’s contracture tissues. The upper part was derived from DD8 without diabetes mellitus (DM), and the lower part was derived from DD7 with DM. **H** Quantification of the S100A4-positive area shown in Fig. 2G and Supplementary Fig. 2. Because of the dense fibrotic matrix, individual cellular boundaries were not clearly distinguishable; therefore, S100A4 expression was quantified as the positive area. The DM (-) and DM (+) groups included nine and nine patients, respectively. Data are shown as the mean ± SEM. The DM (+) group had a significantly larger S100A4-positive area than the DM (-) group (two-tailed Student’s t-test; *p* < 0.001). Scale bars, 50 μm (**C**, **G**).

Consistent with the RNA-seq data, high-glucose treatment upregulated *S100A4* mRNA and protein expression in Dupuytren’s contracture-derived fibroblasts in vitro (Fig. 2A–D, Supplementary Fig. 1D). Extracellular S100A4 levels in the culture medium derived from Dupuytren’s contracture-derived fibroblasts also increased following high-glucose treatment (Fig. 2E). Quantitative real-time polymerase chain reaction (qRT-PCR) analyses using mRNA obtained from Dupuytren’s contracture tissues revealed that *S100A4* expression levels positively correlated with HbA1c levels in blood tests (Fig. 2F). Similarly, immunohistochemical analysis of Dupuytren’s contracture tissues showed that the S100A4-positive area was significantly larger in the DM group than in the non-DM group (Fig. 2G, H, Supplementary Fig. 2). These findings suggest that DM is associated with increased S100A4 expression in Dupuytren’s contracture tissues.

### S100A4 is expressed in stromal cells and macrophages, whereas RAGE and TLR4 are expressed in macrophages

**Fig. 3:**
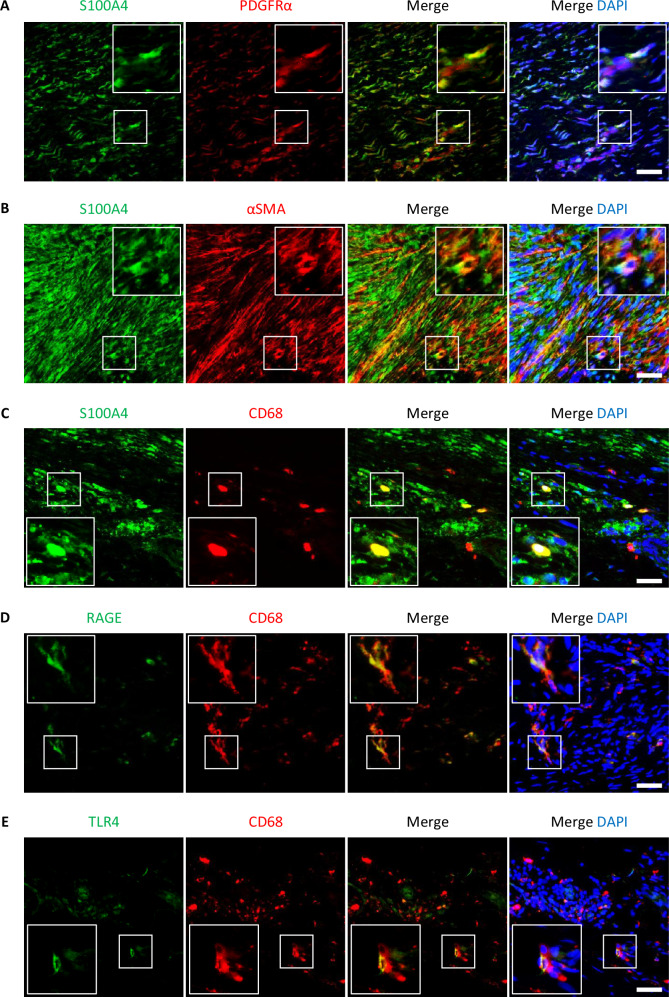
S100A4 is expressed in stromal cells and macrophages, whereas receptor for advanced glycation end-product (RAGE) and Toll-like receptor 4 (TLR4) are expressed in macrophages. **A** Immunofluorescence staining of Dupuytren’s contracture tissues using anti-S100A4 and PDGFRα antibodies. PDGFRα-positive fibroblasts co-expressed S100A4. The white square area is enlarged in the upper-right panel. **B** Immunofluorescent staining of Dupuytren’s contracture tissues using anti-S100A4 and αSMA antibodies. αSMA-positive myofibroblasts co-expressed S100A4. The white square area is enlarged in the upper-right panel. **C** Immunofluorescence staining of Dupuytren’s contracture tissues using anti-S100A4 and CD68 antibodies. CD68-positive macrophages co-expressed S100A4. The white square area is enlarged in the lower left panel. **D**. Immunofluorescence staining of Dupuytren’s contracture tissues using RAGE and CD68 antibodies. CD68-positive macrophages co-expressed S100A4. The white square area is enlarged in the upper-left panel. **E** Immunofluorescence staining of Dupuytren’s contracture tissues using anti-TLR4 and CD68 antibodies. Some CD68-positive macrophages co-expressed TLR4. The white square area is enlarged in the lower left panel. * Scale bars, 50 μm (**A**–**E**).

Next, we investigated S100A4-expressing cells in Dupuytren’s contracture tissues. A Uniform Manifold Approximation and Projection (UMAP) plot was created using the public single-cell RNA sequencing (scRNA-seq) dataset (GSE173252) [21]. *S100A4* expression was broadly observed in several cell types, including *PDGFRA*^+^ (fibroblasts), *ACTA2*^+^ (myofibroblasts), and *CD68*^+^ (macrophages) clusters (Supplementary Fig. 3). Consistent with scRNA-seq data, PDGFRα^+^ fibroblasts, αSMA^+^ myofibroblasts, and CD68^+^ macrophages co-expressed S100A4 (Fig. 3A–C).

Immunohistochemical analysis revealed RAGE expression was detected in a small number of CD68^+^ macrophages and sweat glands (Fig. 3D, Supplementary Fig. 4A). In addition, TLR4 expression was detected in CD68^+^ macrophages and blood vessels (Fig. 3E, Supplementary Fig. 4B). scRNA-seq data revealed that the *CD68*^+^ macrophage cluster highly expressed *TLR4* (Supplementary Fig. 3). In contrast, *AGER* expression was barely observed in any Dupuytren’s contracture-derived cells (Supplementary Fig. 3). These results suggest that macrophages may be a cell type that receives S100A4 primarily via TLR4.

### S100A4 upregulates TGF-β1 expression through TLR4 in macrophages

**Fig. 4:**
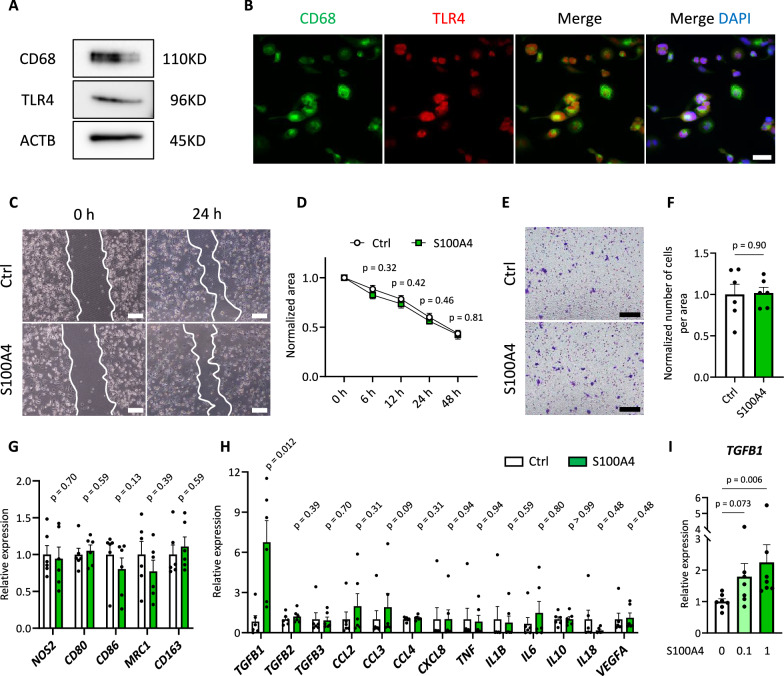
S100A4 upregulates transforming growth factor-beta 1 (TGF-β1) expression through TLR4 in human THP-1 macrophages. **A** Western blot analysis of phorbol myristate acetate (PMA)-treated human THP-1 cells. PMA-treated THP-1 cells expressed CD68 and TLR4, indicating differentiation into macrophages. **B** Immunocytochemical staining using anti-CD68 and TLR4 antibodies in PMA-treated THP-1 macrophages. Most CD68-positive macrophages co-expressed TLR4. **C** The scratch test was used to assess the migration capacity of PMA-treated THP-1 macrophages treated without (control group; Ctrl, upper panel) or with 1 μg/mL recombinant human S100A4 (rhS100A4) protein (S100A4 group; lower panel). Representative images at 0 h (left panel) and 24 h (right panel) are shown. **D** Quantification of the wound-healing rate shown in Fig. 4C (*n* = 4 independent experiments). The mean area at 0 h was set to 1. No significant differences were observed between the two groups (two-tailed Student’s t-test: *p* = 0.32 at 6 h; *p* = 0.42 at 12 h; *p* = 0.46 at 24 h; and *p* = 0.81 at 48 h). **E** Chemotaxis assay of PMA-treated THP-1 macrophages treated with or without 1 μg/mL rhS100A4 protein. The analyses were performed 24 h after seeding. **F** Quantification of the migrated cells shown in Fig. 4E (*n* = 6 independent experiments). The mean normalised cell number in the control group was set to 1. No significant differences were observed between the two groups (two-tailed Student’s t-test, *p* = 0.90). **G** Real-time qPCR analyses comparing macrophage differentiation marker expression in THP-1 macrophages between the control (Ctrl) and S100A4 groups. No significant changes were observed in the M1 and M2 markers (two-tailed Mann–Whitney U test, *n* = 6 independent experiments). **H** Real-time qPCR analyses comparing cytokine and chemokine expression in THP-1 macrophages between the Ctrl and S100A4 groups. *TGFB1* expression was significantly increased in the S100A4 group (*p* = 0.012) (two-tailed Mann–Whitney U test, *n* = 6 independent experiments). **I** Dose-dependent *TGFB1* expression in THP-1 macrophages treated with rhS100A4 protein (0, 0.1 and 1 μg/mL). (Kruskal-Wallis test with Dunn’s multiple comparison test, *n* = 7 independent experiments).

Subsequently, we investigated the function of S100A4 in macrophages in vitro. The human monocyte cell line THP-1 was differentiated into macrophage-like cells using phorbol myristate acetate (PMA). Western blot and immunocytochemistry showed that PMA-treated THP-1 cells expressed TLR4, whereas RAGE expression was barely detectable (Fig. 4A, B, Supplementary Fig. 5A, B). Scratch and chemotaxis assays revealed that recombinant human S100A4 (rhS100A4)-treated THP-1 macrophages showed no significant changes in cell migration or invasion compared to the control (Fig. 4C–F). Gene expression analyses revealed that rhS100A4 did not induce macrophage differentiation into classically activated (M1) or alternatively activated (M2) macrophages; however, significant *TGFB1* upregulation was observed with rhS100A4 treatment (Fig. 4G–I). Similarly, PMA-treated U937 macrophages showed significant upregulation of *TGFB1* expression with rhS100A4 treatment (Supplementary Fig. 6A, B).

### Inhibition of TLR4 suppressed TGF-β1 upregulation induced by S100A4 in macrophages

**Fig. 5:**
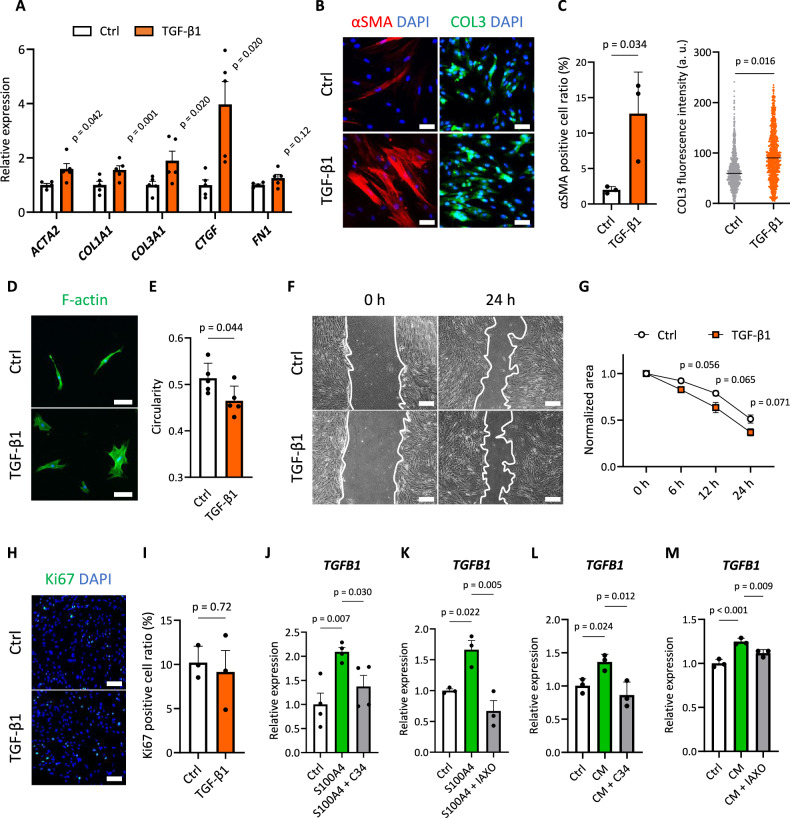
Inhibition of TLR4 suppressed transforming growth factor-beta 1 (TGF-β1) upregulation induced by S100A4 in human THP-1 macrophages. **A** mRNA expression analyses using real-time PCR of recombinant human TGF-β1-treated (1 ng/μL) Dupuytren’s contracture-derived fibroblasts in vitro. Fibrotic markers were significantly upregulated with TGF-β1 treatment (two-tailed paired t-test, *n* = 5 independent biological samples, biological triplicates for each sample). **B** Immunocytochemistry using anti-αSMA and anti-COL3A antibodies for Dupuytren’s contracture-derived cells. The upper panels show control cells without TGF-β1 treatment (Ctrl). TGF-β1 (1 ng/μL) induces myofibroblast differentiation and increases type III collagen expression (lower panels). **C** Quantification of the αSMA-positive cell ratio (*n* = 3 independent experiments, three different fields per experiment) and fluorescence intensity (arbitrary unit; a.u.) of COL3 (*n* = 3 independent experiments, two different fields per experiment) shown in Fig. 5B. TGF-β1 treatment significantly increased the percentage of αSMA-positive cells (two-tailed Student’s t-test, *p* = 0.034) and the expression of COL3 (two-tailed nested t-test, *p* = 0.016). **D** F-actin staining for Dupuytren’s contracture-derived cells. The upper and lower panels show the Ctrl and TGF-β1 groups, respectively. **E** Quantification of cell circularity shown in Fig. 5D (*n* = 5 independent experiments, three different fields per experiment). TGF-β1 treatment significantly increased the cell circularity (two-tailed Student’s t-test; *p* = 0.044). **F** The scratch test was used to assess the migration capacity of Dupuytren’s contracture-derived fibroblasts treated without (Ctrl group; upper panel) or with 1 ng/mL TGF-β1 (TGF-β1 group; lower panel). Representative images at 0 h (left panel) and 24 h (right panel) are shown. **G** Quantification of the wound-healing rate shown in Fig. 5F (*n* = 3 independent experiments). The mean area at 0 h was set to 1. Although no significant differences were observed between the two groups, a trend toward enhanced wound healing was observed in the TGF-β1 group (two-tailed Student’s t-test; *p* = 0.056 at 6 h, *p* = 0.065 at 12 h, and *p* = 0.071 at 24 h). **H** Immunocytochemistry using an anti-Ki67 antibody for Dupuytren’s contracture-derived cells. The upper and lower panels show the Ctrl and TGF-β1 groups, respectively. **I** Quantification of the Ki67-positive cell ratio shown in Fig. 5H (*n* = 3 independent experiments, three different fields per experiment). No significant differences were observed between the two groups (two-tailed Student’s t-test, *p* = 0.72). **J** *TGFB1* expression in THP-1 macrophages treated with 1 μg/mL rhS100A4 protein and 10 μM TLR4-IN-C34 (TLR4 inhibitor). *TGFB1* expression was significantly increased by S100A4 treatment (*p* = 0.007), and the TLR4-IN-C34 significantly suppressed *TGFB1* upregulation induced by S100A4 (*p* = 0.030) (one-way ANOVA with Holm–Sidak multiple comparison test, *n* = 4 independent experiments). **K** *TGFB1* expression in THP-1 macrophages treated with 1 μg/mL rhS100A4 protein and 10 μM IAXO-102 (TLR4 inhibitor). The IAXO-102 significantly suppressed *TGFB1* upregulation induced by S100A4 (*p* = 0.005) (one-way ANOVA with Holm–Sidak multiple comparison test, *n* = 3 independent experiments). **L** *TGFB1* expression in THP-1 macrophages treated with conditioned medium derived from Dupuytren’s fibroblasts and 10 μM TLR4-IN-C34. *TGFB1* expression was significantly increased by conditioned medium (*p* = 0.024), and the TLR4-IN-C34 significantly suppressed *TGFB1* upregulation induced by conditioned medium (*p* = 0.012) (one-way ANOVA with Holm–Sidak multiple comparison test, *n* = 3 independent experiments). **M** *TGFB1* expression in THP-1 macrophages treated with conditioned medium derived from Dupuytren’s fibroblasts and 10 μM IAXO-102. The IAXO-102 significantly suppressed *TGFB1* upregulation induced by conditioned medium (*p* = 0.009) (one-way ANOVA with Holm–Sidak multiple comparison test, *n* = 3 independent experiments). C34: TLR4-IN-C34; IAXO: IAXO-102; *Ctrl* control medium, *CM* conditioned medium. Data are presented as the mean ± SEM. Scale bars: 50 μm (**B**), 100 μm (**D**, **H**), 200 μm (**F**).

TGF-β1 significantly upregulated fibrotic genes, including *ACTA2*, *COL1A1*, *COL3A1*, and *CTGF* (Fig. 5A), and induced activated myofibroblast differentiation and its associated morphological feature in Dupuytren’s contracture-derived fibroblasts (Fig. 5B–E) [22]. TGF-β1-induced myofibroblasts highly expressed αSMA and COL3 and showed increased cell migration, although cell proliferation was unchanged (Fig. 5B, C, F–I). The TLR4 inhibitors TLR4-IN-C34 and IAXO-102 suppressed *TGFB1* upregulation induced by rhS100A4 treatment in THP-1 and U937 macrophages (Fig. 5J, K, Supplementary Fig. 6C, D). Furthermore, these inhibitors suppressed *TGFB1* upregulation induced by conditioned medium derived from Dupuytren’s fibroblasts in macrophages (Fig. 5L, M). These findings suggest that S100A4 regulates TGF-β1 expression in macrophages through TLR4 signalling and induces fibrosis in Dupuytren’s contracture.

## Discussion

In the present study, we investigated the relationship between abnormal glucose metabolism and fibrosis using human Dupuytren’s contracture samples. In contrast to our expectations, RNA-seq analysis revealed that high-glucose treatment of Dupuytren’s contracture-derived fibroblasts decreased the expression of fibrotic markers, including *COL1A1*, *COL1A2*, *POSTN* and *TGFB1*, suggesting that the cell-autonomous fibrotic program may not be activated in stromal cells under high-glucose conditions. Stromal–immune crosstalk plays a pivotal role in the development and progression of various fibrotic diseases, including Dupuytren’s contracture [15–20, 23, 24]. Interactions between IL33-expressing stromal cells and TNF-expressing mast cells and M2 macrophages [16], IL13-expressing mast cells and myofibroblasts [17], collagen IV-expressing myofibroblasts and myeloid cells [20], and CXCL14-expressing stromal cells and M2 macrophages [15] have been identified in Dupuytren’s contracture. Therefore, we hypothesized that stromal cell–immune cell interactions could also be involved in the development and progression of DM-induced Dupuytren’s contracture. In this study, S100A4 expression was significantly increased in Dupuytren’s contracture-derived fibroblasts under high-glucose conditions, and S100A4 upregulated TGF-β1 expression in human THP-1 and U937 macrophages through TLR4 signalling.

The in vitro experiments demonstrated that human THP-1 and U937 macrophages predominantly express TLR4. TLRs are a family of highly conserved receptors that play a fundamental role in regulating early host defences against infections [25]. Additionally, TLRs are involved in non-infectious inflammation by recognising DAMPs, including extracellular matrix components, high-mobility group box 1, heat shock proteins, and S100 family proteins [9, 25]. Extracellular S100A4 regulates differentiation, cytokine production, and apoptosis protection in myeloid cells, especially through the TLR4 signalling pathway [14, 26, 27]. Extracellular S100A4 promotes the M2-like conversion of human primary monocytes and THP-1 cells [27]. S100A4 also promotes IL1β, IL6, and TNF production in peripheral blood mononuclear cells derived from patients with rheumatoid arthritis through TLR4 [14]. However, in contrast to these findings, the present study demonstrated that rhS100A4 treatment did not induce M2-like macrophage polarisation or upregulate *IL1B*, *IL6*, and *TNF* in THP-1 macrophages. Interestingly, S100A4 treatment significantly upregulated *TGFB1* expression in macrophages. TGF-β1 is a major inducer of fibrosis [28], and TGF-β1 treatment upregulates fibrotic gene expression and induces myofibroblast differentiation in human Dupuytren’s contracture-derived fibroblasts [15, 29]. Suga et al. [30] investigated skin wound healing in TLR4-deficient mice and revealed that hyaluronan, a type of DAMP, upregulated TGF-β through TLR4 and thereby regulated wound healing. Similarly, Seki et al. [31] investigated hepatic fibrosis using TLR4-mutant mice and revealed that TLR4 upregulated *Tgfb1* expression and enhanced TGF-β signalling, leading to hepatic fibrosis. Thus, the S100A4–TLR4–TGF-β signalling axis may represent a key mechanism involved in the fibrotic programme in Dupuytren’s contracture caused by aberrant glucose metabolism, and this axis may serve as a novel therapeutic target for Dupuytren’s contracture. Furthermore, appropriate glycaemic control in patients with diabetes may help prevent the development and progression of Dupuytren’s contracture.

However, this study has some limitations that need to be addressed. First, the exact mechanism through which glucose regulates S100A4 expression remains unclear. Although no studies have identified this mechanism, clinical studies have demonstrated that serum S100A4 concentrations are higher in patients with type 2 DM [32] and that plasma S100A4 levels are positively correlated with insulin resistance in prepubertal and pubertal children with obesity [33]. Second, the involvement of RAGE signalling needs to be investigated. RAGE is also a receptor for AGEs [34], indicating it may be closely associated with DM. Notably, RAGE expression was detected in the sweat glands of Dupuytren’s contracture tissues in this study. Sweat glands in Dupuytren’s contracture synthesise CTGF and function as proliferation centres for fibrosis progression [35, 36]. S100A4 may affect the fibrotic programme through sweat glands in Dupuytren’s contracture via cellular crosstalk. Finally, intracellular S100A4 enhances αSMA^+^ myofibroblast trans-differentiation under pathophysiologic conditions mimicking fibrotic stiffness, and loss of intracellular S100A4 fails to induce fibroblast-to-myofibroblast differentiation even in the presence of TGF-β [37]. Thus, intracellular S100A4 may also contribute to the pathogenesis of Dupuytren’s contracture. Further research is required to investigate the mechanism regulating S100A4 expression via glucose and the association of RAGE signalling and the intracellular function of S100A4 in the development and progression of Dupuytren’s contracture.

**Fig. 6:**
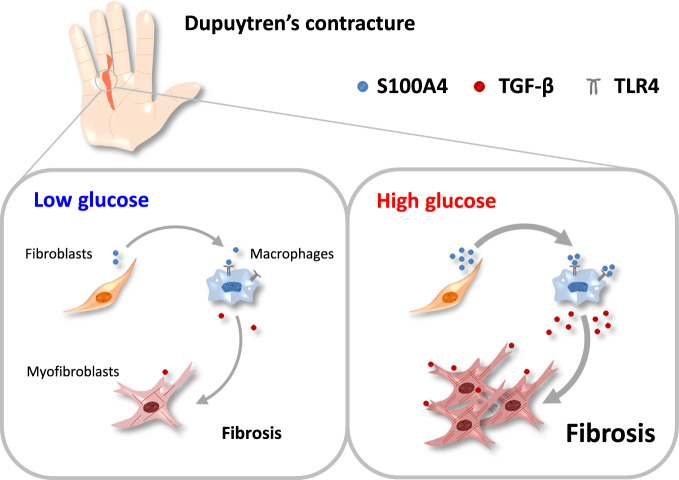
Schematic illustration of fibrotic mechanism through high glucose-induced S100A4–TLR4–TGF-β axis in Dupuytren’s contracture.

In conclusion, this study identified a high glucose-induced fibrotic factor, S100A4, in Dupuytren’s contracture. Fibroblast-expressing S100A4 induced TGF-β1 expression from macrophages through TLR4 signalling, and TLR4 inhibition in macrophages decreased TGF-β1 expression (Fig. 6). Thus, the S100A4–TLR4–TGF-β axis could be a therapeutic target for Dupuytren’s contracture in diabetic patients.

## Materials and methods

### Ethical considerations and study participants

The experiments using clinical samples of human Dupuytren’s contracture were approved by the Ethics Committee of Gifu University (approval number 28-140). Written informed consent was obtained from all patients. All ethical regulations relevant to the human research participants were followed.

Between 2016 and 2025, patients underwent surgery for Dupuytren’s contracture at Gifu University Hospital and were informed of this research. Nineteen patients (18 males and one female) were included (DD1, 72-year-old female; DD2, 86-year-old male; DD3, 76-year-old male; DD4, 76-year-old male; DD5, 71-year-old male; DD6, 75-year-old male; DD7, 51-year-old male; DD8, 80-year-old male; DD9, 71-year-old male; DD10, 86-year-old male; DD11, 75-year-old male; DD12, 70-year-old male; DD13, 79-year-old male; DD14, 55-year-old male; DD15, 60-year-old male; DD16, 53-year-old male; DD17, 71-year-old male; DD18, 71-year-old male; and DD19, 75-year-old male). Surgically excised samples were collected at the time of surgery.

### Cell culture and in vitro experiments

Surgically resected human Dupuytren’s contracture tissues were shredded into 2–3 mm pieces and dissociated with collagenase for 20 min at 37 °C. The cells were cultured in a 10 cm dish (Thermo Fisher Scientific, Waltham, MA, USA) with Dulbecco’s Modified Eagle Medium (DMEM) supplemented with 10% foetal bovine serum (FBS) and 1% penicillin/streptomycin for approximately 2 weeks. Dupuytren’s cells (first passage) were reseeded in 6-, 12-, or 24-well plates. The cells were divided into high-glucose conditions (DMEM low or high glucose with 10% FBS and 1% penicillin/streptomycin; FUJIFILM Wako Pure Chemical Corporation, Osaka, Japan) and cultured for 72 h. The cells were then used for qRT-PCR, western blotting, immunocytochemistry, and RNA sequencing, and the culture supernatant was used for enzyme-linked immunosorbent assay (ELISA). Dupuytren’s cells (first passage) were also treated with 1 ng/mL TGF-β1 (Proteintech, Tokyo, Japan) for 24 h and used for qRT-PCR, migration assay and immunocytochemistry.

The human monocytic leukaemia cell lines THP-1 and U937 were provided by RIKEN BRC (Ibaraki, Japan) and the Japanese Collection of Research Bioresources Cell Bank, National Institutes of Biomedical Innovation, Health and Nutrition (Osaka, Japan), respectively. Both cell lines were maintained in RPMI-1640 medium (FUJIFILM Wako) supplemented with 10% FBS and 1% penicillin/streptomycin. For macrophage differentiation, THP-1 and U937 cells were treated with 200 nM PMA (FUJIFILM Wako) for 3 days. After removing PMA for 2 days, the cells were used for gene expression, migration, and chemotaxis assays. THP-1 and U937 cells were treated with or without 1 μg/mL rhS100A4 protein (R&D Systems, Minneapolis, MN, USA) for 24 h (Fig. 4G–I, Supplementary Fig. 6B). THP-1 and U937 cells were also treated with or without TLR4 inhibitors (10 μM TLR4-IN-C34 and 10 μM IAXO-102 [Selleck Chemicals, Houston, TX, USA]) for 1 h and subsequently with or without 1 μg/mL rhS100A4 protein (R&D Systems) for 24 h (Fig. 5J, K, Supplementary Fig. 6C, D).

For conditioned medium (CM) stimulation experiments, Dupuytren’s contracture-derived fibroblasts were reseeded and cultured in serum-free high-glucose DMEM for 24 h, after which the CM was collected, centrifuged, and passed through a 0.45 µm filter (Sigma-Aldrich, St. Louis, MO, USA). Cell-free medium incubated under the same conditions was used as a control. THP-1 cells were differentiated with PMA and pretreated with TLR4 inhibitors for 1 h before CM treatment. After 24 h of CM stimulation, total RNA was extracted and used for qRT-PCR.

### qRT-PCR

RNA was extracted using the RNeasy Plus Mini Kit (QIAGEN, Venlo, Netherlands). Up to 1 µg of RNA was used for reverse transcription into cDNA using the PrimeScript RT reagent kit (Takara Bio Inc., Kusatsu, Japan). qRT-PCR was performed using Premix Ex Taq™ (Perfect Real Time; Takara Bio Inc). Transcript levels were analysed using three technical replicates and normalised to those of *ACTB*. The number of biological replicates is shown in the Figure legends. The primer sequences are listed in Supplementary Table S1.

### Western blot analysis

Cultured cells were harvested in 150 µL of RIPA lysis buffer, and the protein concentration was measured. Proteins were denatured with 2× sodium dodecyl-sulphate at 95 °C for 5 min, and 20 μg of the denatured protein was loaded onto a 10% sodium dodecyl-sulphate polyacrylamide gel electrophoresis (SDS-PAGE) gel. Separated proteins were transferred onto a polyvinylidene fluoride membrane (Amersham Hybond-P polyvinylidene fluoride membrane; GE Healthcare, Chicago, IL, USA). The membranes were treated with a blocking reagent containing 5% bovine serum albumin (BSA; Sigma-Aldrich) for 1 h at 25 °C. Primary antibodies were applied in Can Get Signal Solution 1 (TOYOBO, Osaka, Japan) overnight at 4 °C and secondary antibodies in Can Get Signal Solution 2 (TOYOBO) for 1 h at 25 °C. Pierce ECL Plus Western Blotting Substrate (Thermo Fisher Scientific) was used for visualisation. Blots were scanned with ImageQuant™ LAS4000 mini4000 (Cytiva, Tokyo, Japan) and quantified using ImageJ software (https://imagej.nih.gov/ij/index.html). The primary antibodies used were anti-S100A4 [D9F9D] (Cell Signalling Technology, Dabcers, MA, USA; dilution 1:1000), anti-CD68 [KP-1] (Abcam, Cambridge, UK; dilution 1:1000), anti-TLR4 [E5D8T] (Cell Signalling Technology; dilution 1:1000), anti-RAGE [EPR21171] (Abcam; dilution 1:1000), and anti-β-actin [13E5] (Cell Signalling Technology; dilution 1:2000); the secondary antibody used was horseradish peroxidase-linked anti-rabbit IgG antibody [#7074] (Cell Signalling Technology; dilution 1:5000).

### ELISA

The concentration of extracellular S100A4 in culture media was quantified using a commercially available human CircuLex S100A4 ELISA Kit Ver.2 (Medical & Biological Laboratories, Tokyo, Japan) according to the manufacturer’s instructions. Briefly, culture media collected from Dupuytren’s contracture-derived fibroblasts were centrifuged to remove cellular debris and stored at −80 °C until analysis. Samples and standards were added to the ELISA plates and incubated as instructed. After washing, the detection antibody and substrate solution were applied sequentially. Absorbance was measured at 450 nm using a microplate reader (Model 680, Bio-Rad). S100A4 concentrations were calculated from a standard curve. All samples were assayed in biological triplicates.

### Migration and chemotaxis assays

Migration and chemotaxis assays were performed as previously described [15]. For the migration assay, PMA-treated THP-1 and U937 macrophages were cultured in 12-well plates. At 100% confluence, they were treated with RPMI-1640 medium (Thermo Fisher Scientific) and supplemented with or without 1 μg/mL rhS100A4 protein (R&D Systems). The bottom of the plates was scratched with a 200 µL micropipette tip and analysed at 0, 6, 12, 24, and 48 h after scratching. Similarly, Dupuytren’s contracture-derived fibroblasts were cultured in 12-well plates with DMEM supplemented with 10% FBS. At 100% confluence, they were treated with or without 1 ng/mL TGF-β1 (Proteintech). The bottom of the plates was scratched with a 200 µL micropipette tip and analysed at 0, 6, 12, and 24 h after scratching.

For the chemotaxis assay, 1.5 × 10^5^ (200 µL) of PMA-treated THP-1 and U937 macrophages were placed into Costar Transwell chambers in 24-well plates (Corning Incorporated, Corning, NY, USA). The bottom wells of the plates were filled with serum-free medium, with or without 1 μg/mL rhS100A4 protein (R&D Systems). The plates were incubated at 37 °C for 24 h. The transwell chamber containing macrophages was washed with PBS, fixed with 2% paraformaldehyde (PFA) for 20 min, and stained with 0.2% crystal violet for 5 min at 25 °C. Nonmigrating cells on the upper side of the membranes were removed by scraping. The membranes were attached to glass slides, and migrating macrophages were counted under a microscope (BX51; Olympus, Tokyo, Japan). The surface area and number of migrating cells were quantified using ImageJ software.

### Histological analysis and immunohistochemistry

All Dupuytren’s tissue samples were fixed overnight in 4% PFA and embedded in paraffin. The samples were sectioned into 5 μm-thick slices. Haematoxylin and eosin and Masson’s trichrome staining were performed using standard protocols. Primary antibodies for immunohistochemistry included anti-S100A4 [D9F9D] (Cell Signalling Technology; dilution 1:200), anti-S100A4 (Clone 922813; R&D Systems; dilution 1:200), anti-αSMA [1A4] (Abcam; dilution 1:200), anti-PDGF receptor α [D1E1E] (Cell Signalling Technology; dilution 1:100), anti-CD68 [KP-1] (Abcam; dilution 1:200), anti-TLR4 [E5D8T] (Cell Signalling Technology; dilution 1:100), and anti-RAGE [EPR21171] (Abcam; dilution 1:100). The samples were incubated with primary antibodies at 4 °C overnight. For 3,3′-diaminobenzidine (DAB) staining, the secondary antibody (Dako EnVision, Dako Japan, Inc., Kyoto, Japan) was applied, and the samples were incubated at 25 °C for 30 min. The stained cells were analysed under a microscope (BX51, Olympus). For immunofluorescence, secondary antibodies were conjugated with Alexa Fluor 488 and Alexa Fluor 594 (Thermo Fisher Scientific), followed by incubation with the cells at 25 °C for 2 h. Nuclei were counterstained with DAPI (Cell Signalling Technology). Stained cells were analysed using a fluorescence microscope (IX83, Olympus).

### Immunocytochemistry

The cultured cells were washed with PBS and fixed in 2% PFA for 15 min at 25 °C. The cells were then treated with a blocking reagent containing 1% BSA (Sigma-Aldrich) for 1 h at 25 °C. Antibodies used for immunocytochemistry included anti-S100A4 [D9F9D] (Cell Signalling Technology; dilution 1:300), anti-CD68 [KP-1] (Abcam; dilution 1:150), anti-TLR4 [E5D8T] (Cell Signalling Technology; dilution 1:150), anti-RAGE [EPR21171] (Abcam; dilution 1:150), anti-PDGF receptor α [D1E1E] (Cell Signalling Technology; dilution 1:200), anti-Ki67 [SP6] (Abcam; dilution 1:150), anti-Collagen III (ab7788; Abcam; dilution 1:300), and anti-αSMA [1A4] (Abcam; dilution 1:300). The samples were incubated with primary antibodies at 4 °C overnight. Secondary antibodies were conjugated with Alexa Fluor 488 and Alexa Fluor 594 (Thermo Fisher Scientific), followed by incubation with the cells at 25 °C for 2 h. Nuclei were counterstained with DAPI (Cell Signalling Technology). Stained cells were analysed using a fluorescence microscope (IX83, Olympus).

### Cell morphological analysis

F-actin staining was performed using Phalloidin-iFluor® 488 (AAT Bioquest, Inc., Pleasanton, CA, USA) according to the manufacturer’s protocol. Briefly, phalloidin was diluted in PBS at a 1:1000 ratio. Dupuytren’s contracture-derived cells, with or without 1 ng/mL TGF-β1 (Proteintech), were stained with phalloidin for 1 h at 25 °C. The stained cells were rinsed with PBS three times and analysed using a fluorescence microscope (IX83, Olympus). Cell circularity was quantified using ImageJ software based on a previous article [22].

### RNA-sequencing

Dupuytren’s contracture-derived fibroblasts from two patients (DD13 and DD16) were cultured for 72 h (biological triplicates for each sample). RNA was extracted using the RNeasy Plus Mini Kit (QIAGEN). The integrity of the isolated RNA was verified using 2100 Expert Eukaryote Total RNA Nano (Agilent Technologies, Santa Clara, CA, USA). The RNA integrity values of the samples were 9.7–10. DEGs were identified using the exact test after normalisation. The RNA sequencing data were analysed using iDEP 2.01 (http://bioinformatics.sdstate.edu/idep/).

### Statistical analyses

Statistical analyses were performed using GraphPad Prism 10.5.0 (GraphPad Software, La Jolla, CA, USA). All quantitative data are shown as the mean ± standard error of the mean (SEM). The sample sizes and counting procedures were reported in each parametric two-tailed Student’s t-test, nonparametric two-tailed Mann–Whitney U test, one-way ANOVA test, and Pearson correlation coefficient test. Differences were considered significant at *p* < 0.05.

## Supplementary information

Supplementary Figures and Tables

Original data

## Source data

Source data

## Data Availability

The source data for the graphs are available as source data files, and the uncropped blots are provided in Supplementary Fig. 7. The RNA sequencing data were deposited in the Gene Expression Omnibus (GEO) database under accession code (GSE304250).
