# Supplementary material for: S100A4–TLR4–TGF-β axis as a therapeutic target for Dupuytren’s contracture in diabetic patients
Source: Cell Death Discov. 2026 May 23;12:310. doi: 10.1038/s41420-026-03167-y (PMC13376557; doi:10.1038/s41420-026-03167-y)
Supplement: Supplementary file 1 — Supplementary Figures and Tables [file 41420_2026_3167_MOESM1_ESM.pdf]

Supplementary information

S100A4–TLR4–TGF-β axis as a therapeutic target for Dupuytren's contracture in diabetic patients

Authors: Koki Kato[1,‡], Shingo Komura[1,‡,*], Yuta Yanagihara[2], Noritaka Saeki[3], Atsushi Goto[1], Rie Maki[1], Hitoshi Hirose[1], Akihiro Hirakawa[1], Yuuki Imai[2], Haruhiko Akiyama[1,4]

Affiliations:
[1] Department of Orthopaedic Surgery, Gifu University Graduate School of Medicine, Gifu, Japan
[2] Division of Integrative Pathophysiology, Proteo-Science Center, PIAS, Ehime University, Ehime, Japan
[3] Research Coordination and Technical Development Office, PIAS, Ehime University, Ehime, Japan
[4] Center for One Medicine Innovative Translational Research (COMIT), Gifu University, Gifu, Japan

‡These authors contributed equally to this work

***Correspondence**
Shingo Komura, MD, PhD
Department of Orthopaedic Surgery
Gifu University Graduate School of Medicine
1-1 Yanagido, Gifu 501-1194, Japan.
Email: komura.shingo.m7@f.gifu-u.ac.jp
Tel: +81-58-230-6333
Fax: +81-58-230-6334

Supplementary Information
Legends to Supplementary Figures (Supplementary Fig. 1-7)
Legend to Supplementary Table 1

Supplementary Fig. 1

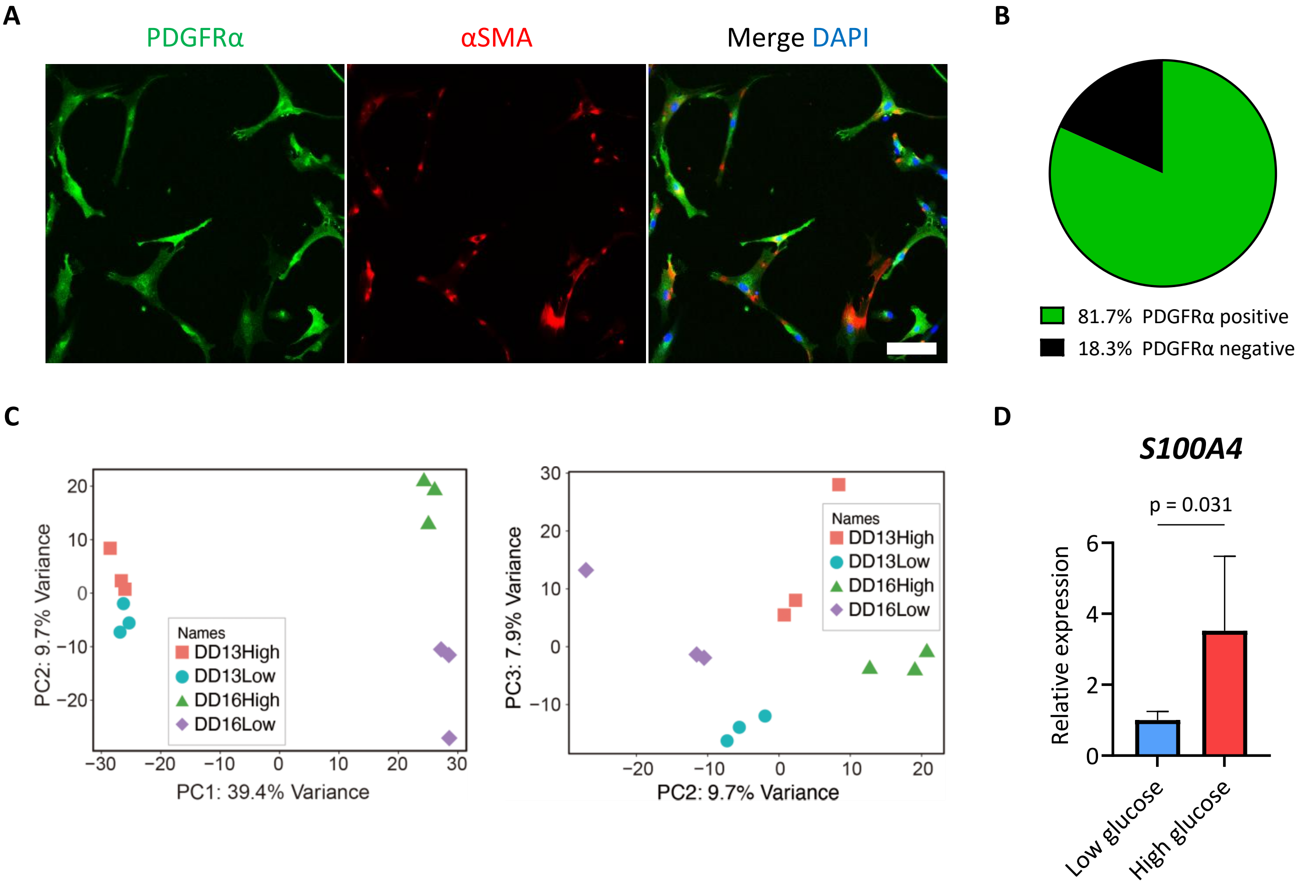

Supplementary Fig. 1

RNA sequencing analysis for human Dupuytren's contracture-derived fibroblasts

A. Immunohistochemical staining of Dupuytren's contracture-derived cells using anti-PDGFRα (green) and αSMA (red) antibodies. Most cells expressed fibroblast-marker PDGFRα, and some cells co-expressed the myofibroblast-marker αSMA (scale bars, 100 µm).

B. The PDGFRα-positive cell ratio of Dupuytren's contracture-derived cells in vitro was 81.7%.

C. Principal component analysis (PCA) was used to visualise sample-to-sample variation. In the left panel, the two components PC1 and PC2 explain 39.4% and 9.7% of the variability in the expression data, respectively. In the right panel, the two components PC2 and PC3 explain 9.7% and 7.9% of the variability in the expression data, respectively.

D. Real-time qPCR analyses comparing *S100A4* expression in human Dupuytren's contracture-derived cells between the low and high glucose conditions. S100A4 expression was significantly increased in the high glucose condition (p = 0.031) (two-tailed Wilcoxon matched-pairs signed rank test, n = 6 independent samples). Transcript levels were analysed using three technical replicates and normalised to those of *GAPDH*.

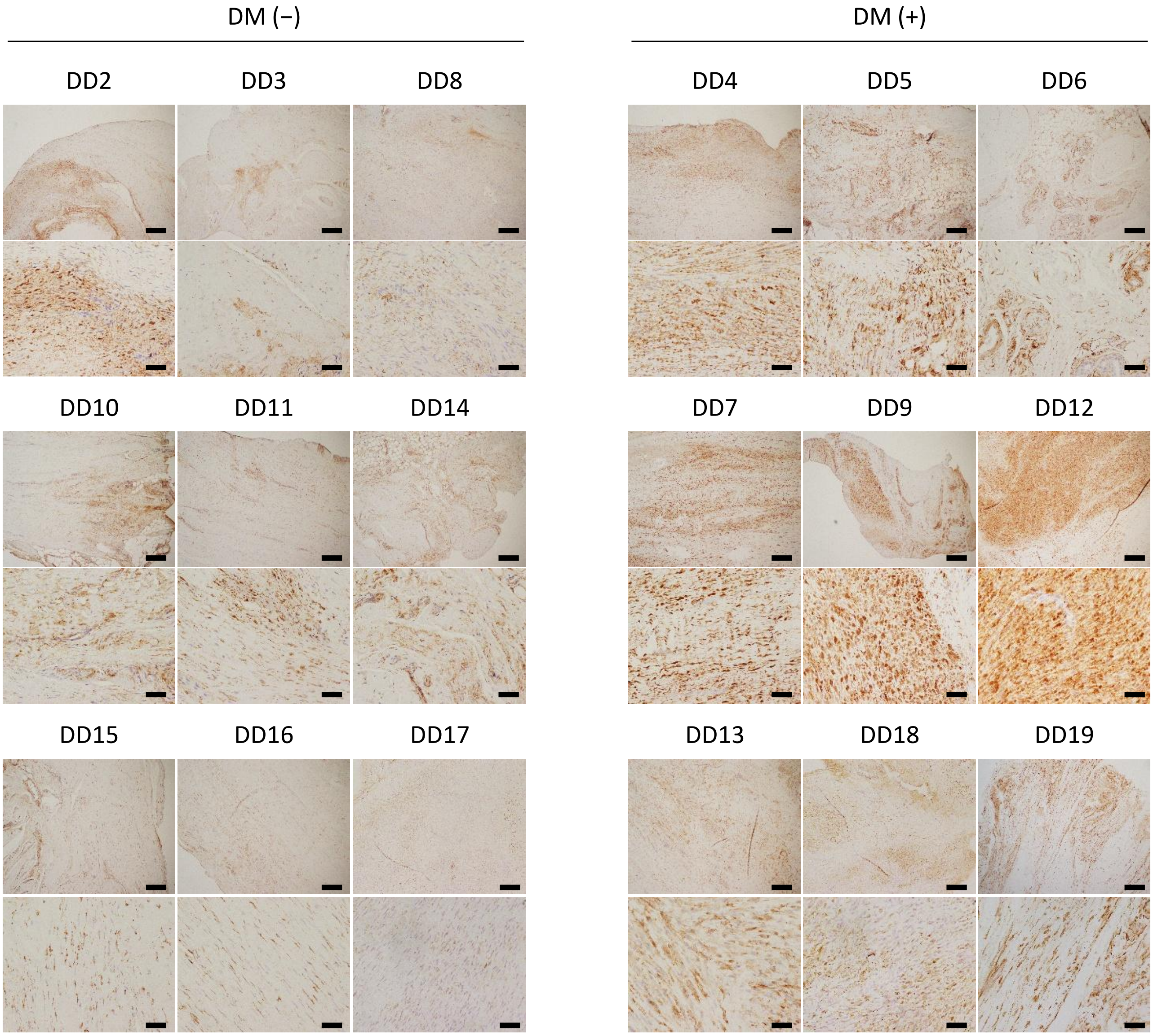

Supplementary Fig. 2

S100A4 expression in Dupuytren's contracture tissues

S100A4 immunohistochemical staining of Dupuytren's contracture tissues (DD2 to DD19). The left group comprised patients without diabetes mellitus (DM) (DD2, 3, 8, 10, 11, 14, 15, 16, and 17), and the right group comprised those with DM (DD4, 5, 6, 7, 9, 12, 13, 18, and 19). The top panels show lower magnification (scale bars, 200 µm) and the bottom panels show higher magnification (scale bars, 50 µm).

Supplementary Fig. 3

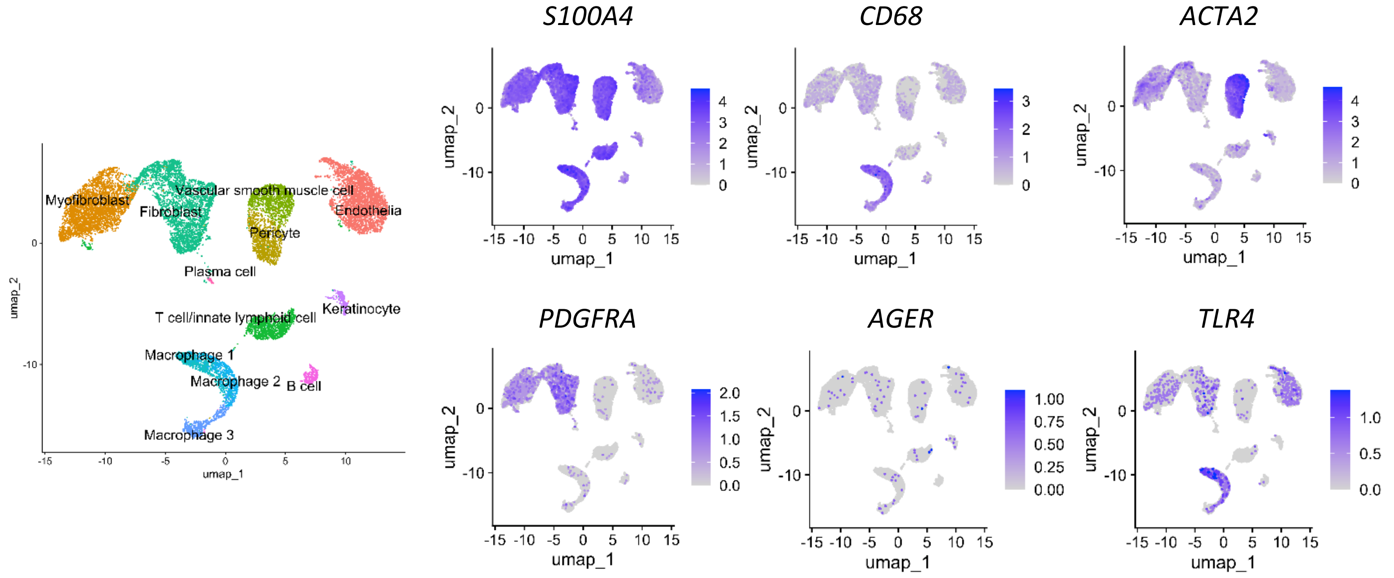

Supplementary Fig. 3
Uniform Manifold Approximation and Projection (UMAP) plot from public human Dupuytren's contracture single cell RNA-sequencing data
A public single-cell RNA sequencing dataset (GSE173252) deposited by Dobie et al. [21] was used to create UMAP plots. *S100A4* expression was observed in the *CD68*$^{+}$ (macrophages), *PDGFRA*$^{+}$ (fibroblasts), and *ACTA2*$^{+}$ (myofibroblasts) clusters. Strong *TLR4* expression was observed in macrophage clusters, whereas low *AGER* expression was observed in each cluster. The analysis method is presented in the source data file (Source data for Supplementary Fig. 3).

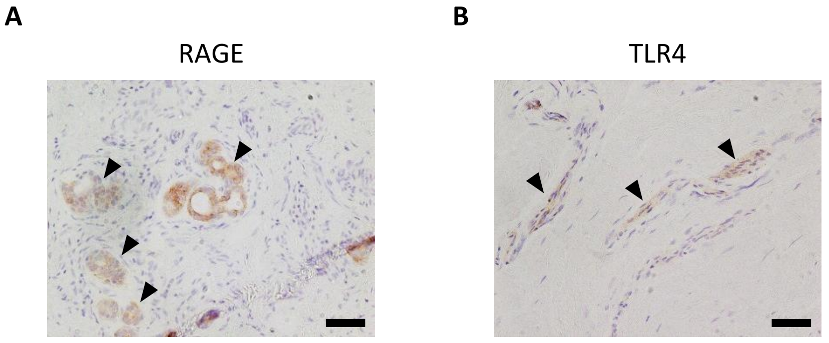

Supplementary Fig. 4
Receptor for advanced glycation end product (RAGE) and Toll-like receptor 4 (TLR4) expression in Dupuytren's contracture tissues
Immunohistochemical staining of Dupuytren's contracture tissues using anti-RAGE and TLR4 antibodies.
A. RAGE expression was detected in the sweat glands (black arrowheads) (left).
B. TLR4 expression was detected in the vessels (black arrowheads) (right).
Scale bars, 50 µm (A, B)

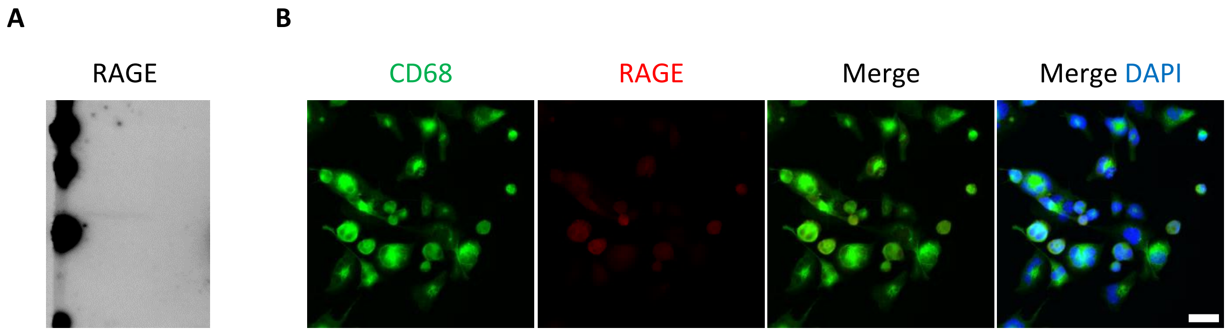

Supplementary Fig. 5

RAGE expression in phorbol myristate acetate-treated human THP-1 macrophages

A. Western blot analysis of phorbol myristate acetate (PMA)-treated human THP-1 macrophages showed low RAGE expression.

B. Immunocytochemical staining using anti-CD68 and RAGE antibodies for PMA-treated THP-1 macrophages. CD68-positive macrophages weakly co-expressed RAGE (scale bar, 50 µm).

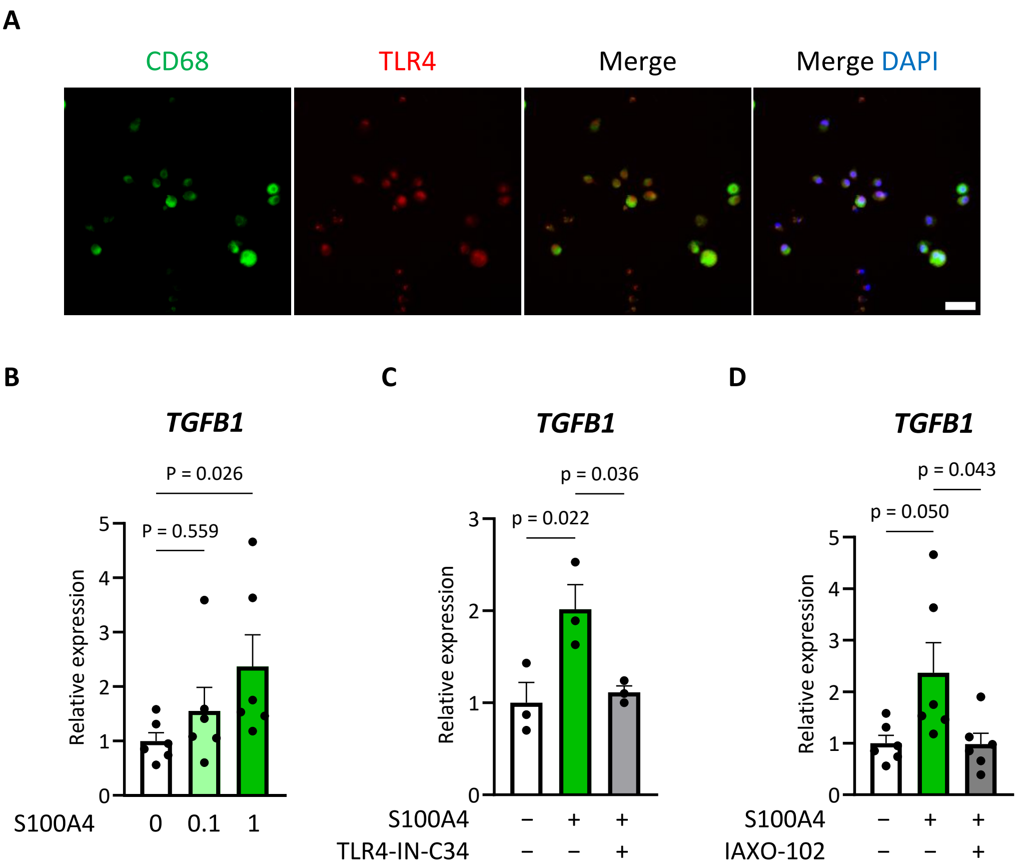

Supplementary Fig. 6

S100A4 upregulates TGF-β1 expression through TLR4 in phorbol myristate acetate-treated human U937 macrophages

A. Immunocytochemical staining using anti-CD68 and TLR4 antibodies for phorbol myristate acetate-treated U937 macrophages. CD68-positive macrophages co-expressed TLR4 (scale bar, 50 µm).

B. Dose-dependent *TGFB1* expression in U937 macrophages treated with recombinant human S100A4 (rhS100A4) protein (0, 0.1 and 1 µg/mL). 1 µg/mL rhS100A4 significantly increased *TGFB1* expression ($p = 0.026$) (Kruskal-Wallis test with Dunn's multiple comparison test, n = 6 independent experiments).

C. *TGFB1* expression in U937 macrophages treated with 1 µg/mL rhS100A4 protein and 10 µM TLR4-IN-C34 (TLR4 inhibitor). *TGFB1* was significantly increased via S100A4 treatment ($p = 0.022$), and the TLR4-IN-C34 significantly suppressed *TGFB1* upregulation by S100A4 ($p = 0.036$) (one-way ANOVA with Dunnett's multiple comparison test, n = 3 independent experiments).

D. *TGFB1* expression in U937 macrophages treated with 1 µg/mL rhS100A4 protein and 10 µM IAXO-102 (TLR4 inhibitor). The IAXO-102 significantly suppressed *TGFB1* upregulation by S100A4 ($p = 0.043$) (Kruskal-Wallis test with Dunn's multiple comparison test, n = 6 independent experiments).

Supplementary Fig. 7
Images of the full membrane supporting Fig. 2A, Fig. 4A, and Supplementary Fig. 5A

Fig. 2A

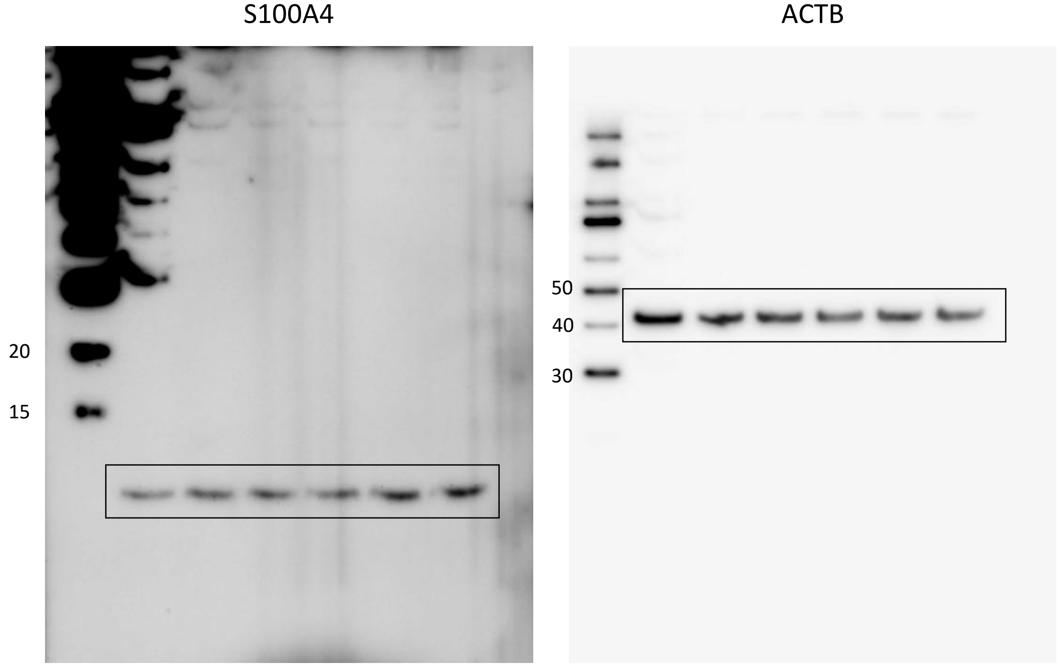

Fig. 4A and Supplementary Fig. 5A

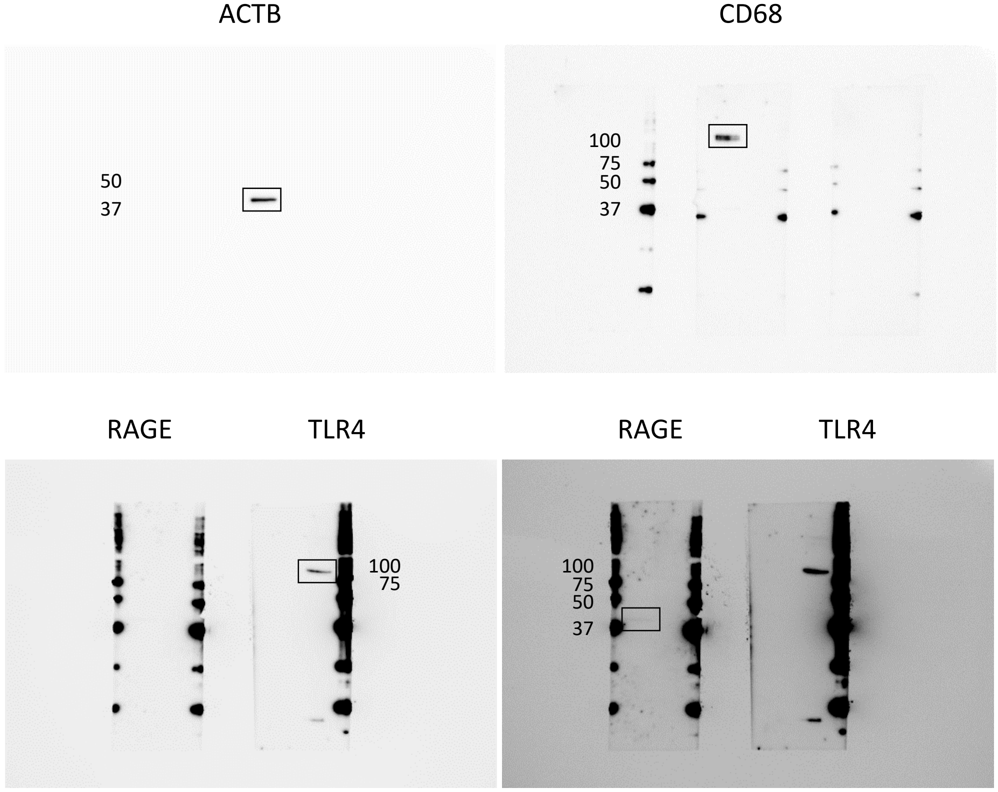

Supplementary Table 1. Primer sequences used in this study

		Genes	Forward (5′ ⇒ 3′)	Revers (5′ ⇒ 3′)
Human	Real-time PCR	*ACTB*	AGACCTGTACGCCAACACAG	GGAGCAATGATCTTGATCTTCA
		ACTA2	ACCGAATGCAGAAGGAGATCAC	CCGATCCAGACAGAGTATTTGCG
		ARG1	TCCTTTCTCAAAGGGACAGC	CATTAGGGATGTCAGCAAAGG
		CCL2	TCAGCCAGATGCAATCAATG	AGCTTCTTTGGGACACTTGC
		CCL3	TGCAACCAGTTCTCTGCATC	GCTTGGTTAGGAAGATGACACC
		CCL4	TGCTAGTAGCTGCCTTCTGC	GCTTGCTTCTTTTGGTTTGG
		CD80	CACTTCTGTTCAGGTGTTATCCAC	TCCCCAGACATCATAGTCAGC
		CD86	GCATTTGTGACAGCACTATGG	GCAGGTCTGCAGTCTCATTG
		CD163	TGAAGACTCTGGATCTGCTGAC	TCAGCTCCTTGTCTGTTCCTC
		COL1A1	ACTGGTGAGACCTGCGTGTAC	TCGAACTGGAATCCATCGGTC
		COL1A3	TGGGGAAACATGCATAAGTG	GCTAAACTGAAAACCACCATCC
		CTGF	GTGTGCACCGCCAAAGAT	AGGCACGTGCACTGGTACTT
		CXCL8	GTGTGAAGGTGCAGTTTTGC	TGTGGTCCACTCTCAATCACTC
		FN1	AGCAAGCCCGGTTGTTATG	GCAAGTCTCTTCAGCTTCAGG
		GAPDH	ATGGGGAAGGTGAAGGTCG	GGGGTCATTGATGGCAACAATA
		IL1B	GCCAATCTTCATTGCTCAAG	GGCCATCAGCTTCAAAGAAC
		IL6	TGAACTCCTTCTCCACAAGC	GCGGCTACATCTTTGGAATC
		IL10	GAGATGCCTTCAGCAGAGTG	CCCAGGTAACCCTTAAAGTCC
		IL18	CAGGAATAAAGATGGCTGCTG	GGTCAATGAAGAGAACTTGGTC
		MRC1	TGCTGTTCTCCTACTGGACAC	ATTTCTGTGATTCGGCATCC
		S100A4	TTCCACAAGTACTCGGGCAAAG	TGGAAAGCAGCTTCATCTGTCC
