# Supplementary figures and images for: S100A4–TLR4–TGF-β axis as a therapeutic target for Dupuytren’s contracture in diabetic patients
Source: Cell Death Discov. 2026 May 23;12:310. doi: 10.1038/s41420-026-03167-y (PMC13376557; doi:10.1038/s41420-026-03167-y)

Fig. 2A

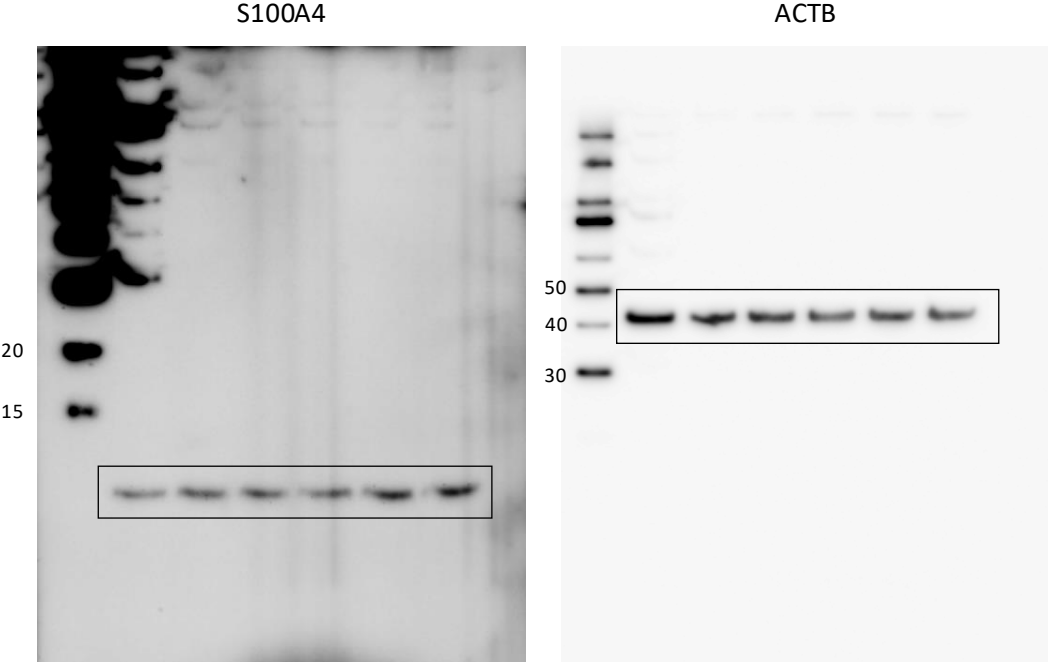

Fig. 4A and Supplementary Fig. 5A

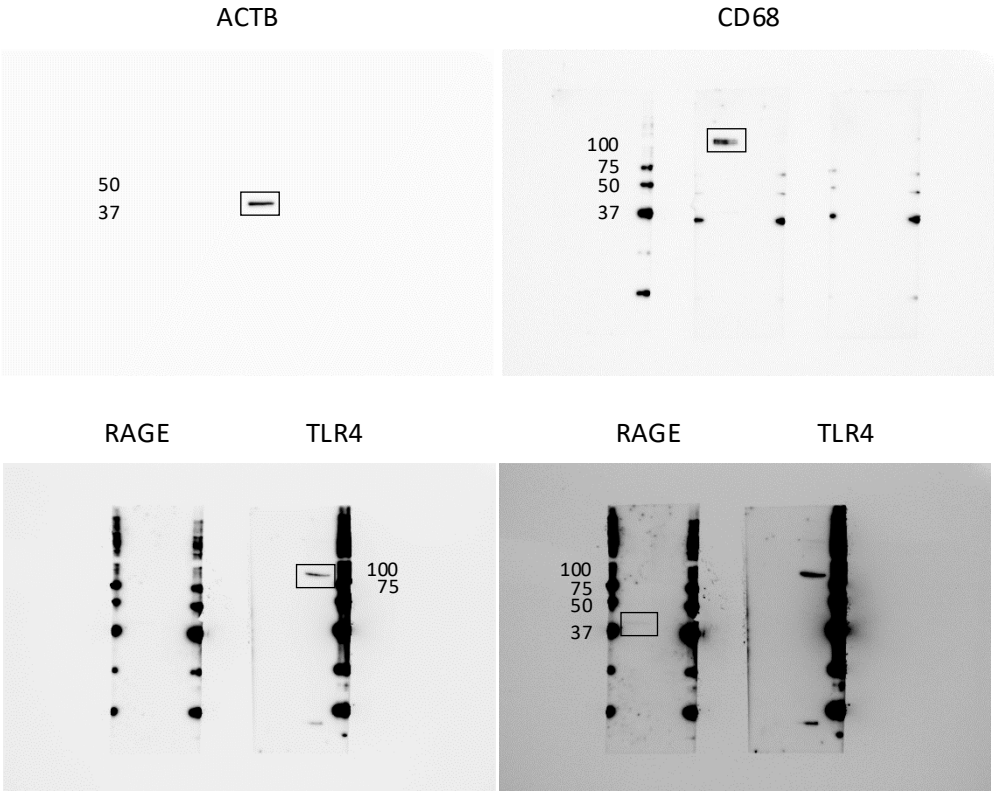

Supplement: Supplementary file 2 — Original data [file 41420_2026_3167_MOESM2_ESM.pdf]
